# Personals

**Published:** 1913-11

**Authors:** 


### PERSONALS.

*Announcements of removal, travel, and other matters of interest are requested. Please report errors in the listing of any physician in the State and other directories that we may co-operate with the State Society in securing a correct list.*

Dr. Lesser Kauffman of Buffalo announces the removal of his office and residence to 534 Elmwood avenue.

Dr. W. L. Phillips of Buffalo is in Europe.

Dr. A. J. Bennett of Busti is chairman of the Democratic Committee of Chautauqua County.

Dr. Edward W. Janes of Tacoma, Wash., has moved his office to 303 S. Ninth street.

Dr. John H. Pryor of Buffalo returned late in October from a European trip of several months.

Dr. George W. MacPherson of Lancaster has moved to Los Angeles.

Burglars visited the residence of Dr. N. W. Wilson of 822 Richmond avenue, Buffalo, October 13. The following articles were among those taken: Black sealskin purse, with money, silver mounted pencil, revolver, ruby stick pin with twelve small diamonds, gold diamond stickpin.

Lt. Col. Eugene A. Smith of Saranac Lake, formerly of Buffalo, has changed his local address to 46 Franklin avenue.

